# Value of Lung Ultrasonography in the Diagnosis and Outcome Prediction of Pediatric Community-Acquired Pneumonia with Necrotizing Change

**DOI:** 10.1371/journal.pone.0130082

**Published:** 2015-06-18

**Authors:** Shen-Hao Lai, Kin-Sun Wong, Sui-Ling Liao

**Affiliations:** 1 Department of Pediatrics, Chang Gung Memorial Hospital, Taoyuan Taiwan; 2 Department of Pediatrics, Chang Gung Memorial Hospital, Keelung, Taiwan; 3 Department of Pediatrics, Chang Gung University, Taoyuan Taiwan; University of California, Merced, UNITED STATES

## Abstract

**Background:**

Lung ultrasonography has been advocated in diagnosing pediatric community-acquired pneumonia. However, its function in identifying necrotizing pneumonia, a complication, has not been explored. This study investigated the value of lung ultrasonography in diagnosing pediatric necrotizing pneumonia and its role in predicting clinical outcomes.

**Methods:**

We retrospectively reviewed 236 children with community-acquired pneumonia who were evaluated using lung ultrasonography within 2–3 days after admission. The ultrasonographic features assessed included lung perfusion, the presence of hypoechoic lesions, and the amount of pleural effusion. Chest computed tomography was also performed in 96 patients as clinically indicated. Detailed records of clinical information were obtained.

**Results:**

Our results showed a high correlation between the degree of impaired perfusion in ultrasonography and the severity of necrosis in computed tomography (*r* = 0.704). The degree of impaired perfusion can favorably be used to predict massive necrosis in computed tomography (area under the receiver operating characteristic curve, 0.908). The characteristics of impaired perfusion and hypoechoic lesions in ultrasonography were associated with an increased risk of pneumatocele formation (odds ratio (OR), 10.11; 95% CI, 2.95–34.64) and the subsequent requirement for surgical lung resection (OR, 8.28; 95% CI, 1.86–36.93). Furthermore, a longer hospital stay would be expected if moderate-to-massive pleural effusion was observed in addition to impaired perfusion in ultrasonography (OR, 3.08; 95% CI, 1.15–8.29).

**Conclusion:**

Lung ultrasonography is favorably correlated with chest computed tomography in the diagnosis of necrotizing pneumonia, especially regarding massive necrosis of the lung. Because it is a simple and reliable imaging tool that is valuable in predicting clinical outcomes, we suggest that ultrasonography be applied as a surrogate for computed tomography for the early detection of severe necrotizing pneumonia in children.

## Introduction

Community-acquired pneumonia (CAP) is among the most common causes of hospitalization in children. Complications of pediatric CAP include parapneumonic effusion, empyema, abscess, and necrotizing changes in lung parenchyma (necrotizing pneumonia, NP). The pathogenesis of NP is associated with the devitalization of lung tissue due to infection. *Staphylococcus aureus*, *Mycoplasma pneumoniae*, and especially, *Streptococcus pneumoniae* can all cause NP [1]. Despite a favorable long-term outcome following pediatric NP, serious morbidity and prolonged hospitalization can inevitably increase medical costs considerably [1–5].

NP is a severe complication of pneumonia, in which the inflamed lung tissue becomes necrotic. After the liquefaction and absorption of the necrotic tissue, a pneumatocele or bronchopleural fistula may develop. Although NP can be suspected because of the presence of thin-walled cavitation (pneumatocele) or pleural free air (bronchopleural fistula) in plain chest radiography, chest computed tomography (CT) remains the most sensitive and accurate tool for definitive diagnosis [6–8]. However, the limitations of CT scanning, such as radiation exposure, high cost, and inconvenient availability in some areas, would discourage its early usage for the prompt detection of necrotizing changes.

Lung ultrasonography (LUS) is a feasible, portable, and nonionizing radiation technique that has been used for diagnosing pleural and pulmonary diseases in children [9–14]. Compared with plain radiography and CT, LUS exhibited high sensitivity and specificity in the diagnosis of pediatric CAP [13, 15]. Recently, LUS has been advocated as a useful tool for detecting pneumonia in the pediatric population [10, 16]. Although few studies describe the sonographic characteristics of NP [17, 18], none have investigated the correlation of LUS with CT imaging and its practicality in diagnosing NP.

The primary aim of this study was to compare the accuracy of LUS with that of CT imaging, which is the gold standard in the assessment of necrotic changes in pediatric CAP. Furthermore, we evaluated the role of LUS in predicting the outcomes of children with NP.

## Materials and Methods

### Patient Population

The design and process of this retrospective study were approved by the institutional review board of Chang Gung Memorial Hospital. Inpatient children admitted because of CAP, in the period of January 2008 to December 2012, and having LUS performed within 2–3 days of admission were recruited for the retrospective review. The CAP diagnosis was based on a clinical examination and chest radiography. Patients with coexisting lung diseases and predisposing congenital abnormalities were excluded. In addition, the medical records and imaging findings on all patients were reviewed. All patient records and clinical information were anonymized and deidentified prior to analysis. The individuals in this manuscript have provided written informed consent (as outlined in the PLOS consent form) for publishing their case details.

For comparing the accuracy of LUS with that of CT, the enrollees who received chest CT as clinically indicated were further enrolled for the analysis of concordance. To minimize the predisposition, only cases who underwent CT within 3 days after LUS examination were enrolled for the comparison. To determine the value of LUS in predicting clinical outcomes, all enrollees were evaluated.

### Lung ultrasonography technique

All ultrasonographies were performed before CT scan examinations. LUS was performed by a single experienced pediatric pulmonologist (S.H.L.) using an Acuson TX 128 machine (Acuson, Mountain View, CA) equipped with curved (5–8 MHz and 2–5 MHz) and linear (5–12 MHz) transductors. Anterior, posterior, subaxillary, and subcostal images were obtained from both transverse and longitudinal sections by using an intercostal approach. The interpretation of ultrasonography was blind to the CT finding. Hypoechoic lesions (HLs) observed in LUS were defined as rough-contoured heterogeneously hypoechoic areas in the consolidated lung.

**Fig 1:**
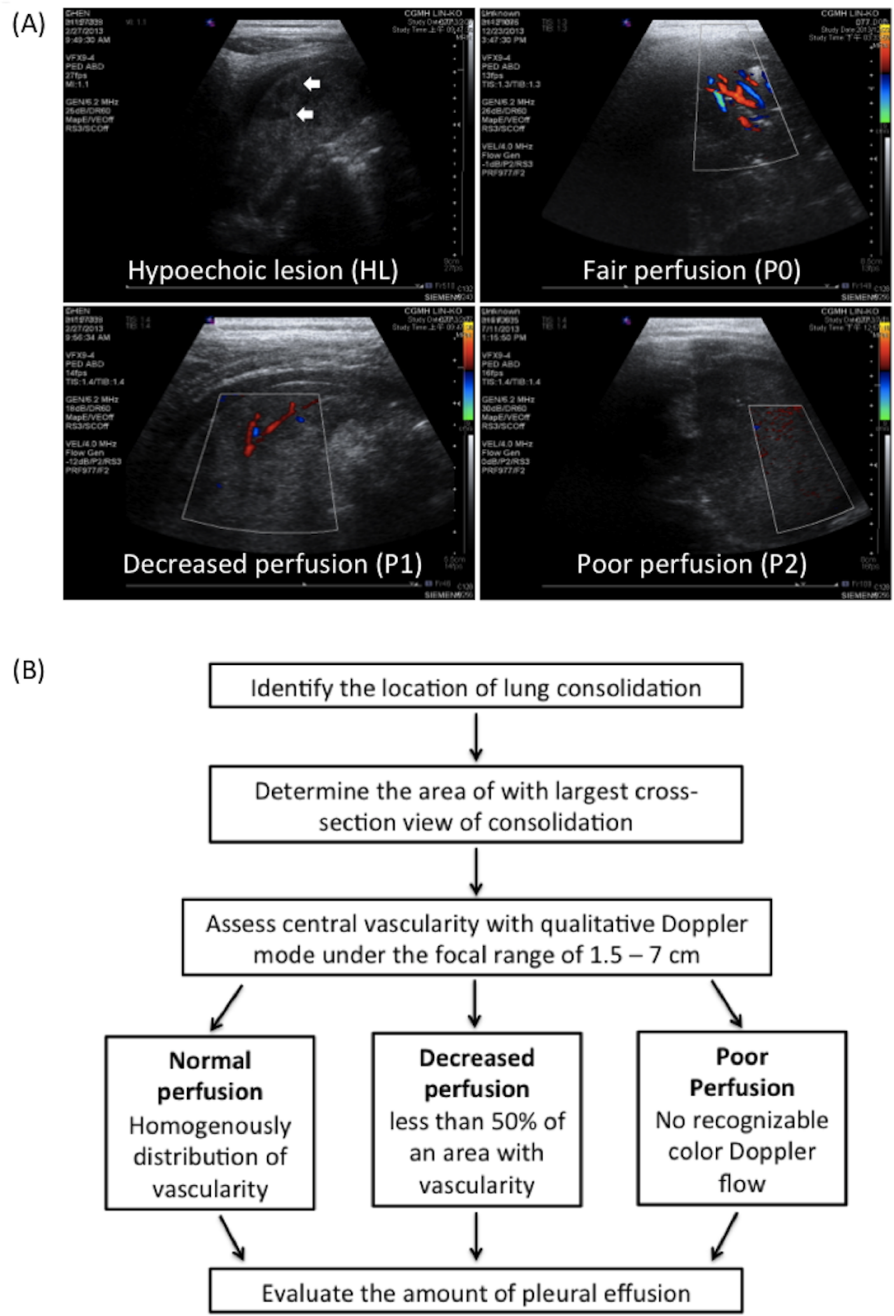
Ultrasonographic grading of pneumonia with necrotizing changes and flow chart of ultrasonograhic assessment. (A) Hypoechoic lesions (HLs), rough-contoured heterogeneously hypoechoic areas in the consolidated lung, are indicated with arrows. Perfusion within the consolidated lung was assessed according to vascularity by using color Doppler. Normal, decreased, and poor perfusion are designated with P0, P1, and P2. (B) Step-by-step flow chart of ultrasonographic assessment.

Enhanced, tree-like vascularity extending from the center to the periphery is a characteristic feature of the consolidated parenchyma in pneumonia [19]. Perfusion of the consolidated lung was assessed within its largest cross-sectional view. Linear probe (5–12 MHz) with qualitative color Doppler mode was used under a focal depth of 1.5 to 7 cm. The perfusion of consolidated lung was classified into various degrees of central vascularity based on the qualitative assessment of the area of color flow as follows (Fig 1A):

1. Normal perfusion (P0): defined as homogenously distributed tree-like vascularity.
2. Decreased perfusion (P1): defined as less than 50% of an area with typical tree-like vascularity.
3. Poor perfusion (P2): defined as no recognizable color Doppler flow.

The amount of pleural effusion in patients in the supine position was determined by measuring the thickness of fluid content at the costophrenic angle in the posterior subaxillary area. A fluid thickness of less than 1 cm was considered minimal; between 1 to 2 cm was considered moderate; and a thickness of more than 2 cm was considered massive. A flow chart of step-by-step ultrasonographic assessment was shown in Fig 1B.

### Chest computed tomography and diagnosis of necrotizing pneumonia

**Fig 2:**
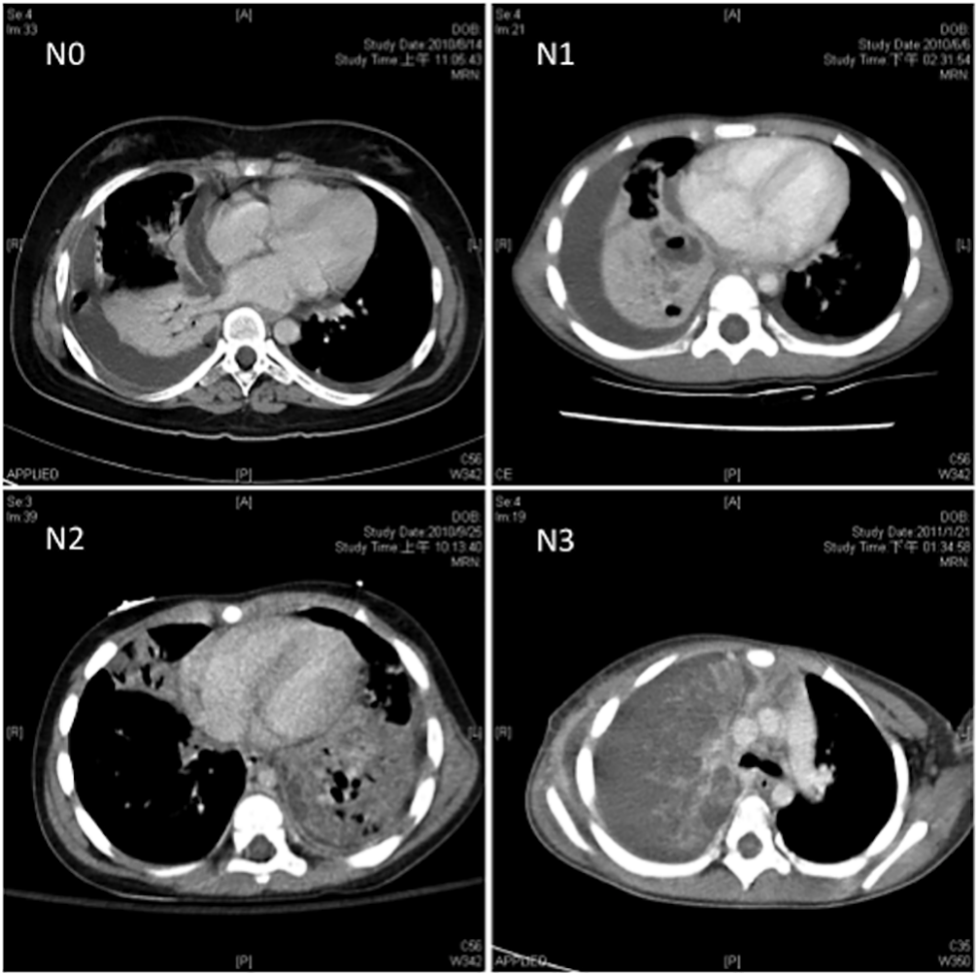
Computed tomographic grading of pneumonia with necrotizing changes. Severity of lung necrosis was measured on the basis of the absence of contrast uptake in computed tomography. Necrotic areas that were 0%, less than 30%, between 30 and 80%, and more than 80% were further categorized into no (N0), mild (N1), moderate (N2), and massive (N3) necrosis.

NP was diagnosed on the basis of contrast-enhanced chest CT images or discrete pneumatoceles showing on serial plain films. The CT findings on NP included the following: (1) a consolidated area without loss of volume, (2) a necrotic radiolucent image within the consolidated area, and (3) the lack of contrast enhancement in CT after contrast administration. The severity of lung necrosis was determined by analyzing the relative ratio of the necrotic radiolucent space to the total consolidated lung area calculated using ImageJ, Version 1.47 (National Institute of Health, USA). This novel approach was a modification of the method for estimating the volume fraction of acute lobar nephronia [20]. Necrotic areas were categorized as mild (N1) if the ratio was less than 30%; moderate (N2) if the ratio was between 30% and 80%; and massive (N3) if the necrotic area was more than 80% (Fig 2). Pneumatoceles were defined as cysts, which are air-filled spaces with sharply demarcated thin walls, formed during the resolution of pneumonia. Bronchopleural fistula was confirmed by the presence of pneumothorax on radiographic images, persistent air leakage from chest tubes, or visualization during surgical intervention.

### Statistical analysis

Comparisons between the groups were performed using Student’s *t* test for continuous variables and Fisher’s exact test for categorical variables. The correlation between LUS and CT features was assessed using the chi-squared test and Spearman correlation analysis. To determine whether the impairment of perfusion was predictive of the varying severity of necrosis in NP, a receiver operating characteristic curve was also obtained. The logistic regression model was used to estimate ORs and 95% confidence interval of the clinical predictive value of LUS. A P value of < 0.05 was considered statistically significant. All analyses were performed using IBM SPSS software, Version 20.

## Results

### Demographic data of participants

A total of 236 children with CAP were evaluated using LUS within 2–3 days of admission. In total, NP was diagnosed in 80 patients with CAP (LUS group, 10/140; LUS + CT group, 70/96). Chest CT was also performed in 96 patients as clinically indicated, mostly within 5 days of admission (Table 1). Thus, apparently, the patients who underwent LUS plus CT (LUS + CT group) exhibited more severe initial presentations, and consequently, had a higher incidence of pneumatocele formation and a higher risk of requiring subsequent surgical intervention with longer hospitalization. The occurrence of bronchopleural fistula was also higher in the LUS + CT group.

**Table 1:** Demographic data and clinical outcomes of participants.

Characteristics	LUS (n = 140)	LUS + CT (n = 96)	All (n = 236)	*p*-value *
**Age**	5.53 ± 3.62	4.88 ± 4.13	5.26 ± 3.84	0.202
**Male gender**	75 (53.6)	44 (45.8)	119 (50.4)	0.289
**Pathogens** ^#^ **(%)**	22/42/33/0/2	14/72/6/2/6	19/54/22/1/4	< 0.001
**Initial presentations**				
Leukocyte, × 10^9^/L	14.3 ± 9.5	16.7 ± 8.8	15.3 ± 9.3	0.048
CRP, mg/dL	168 ± 111	227 ± 114	192 ± 116	< 0.001
Coagulopathy	12/140 (8.6)	28/96 (29)	40/236 (17)	< 0.001
Shock	4/140 (2.6)	6/96 (6.3)	10/236 (4.2)	0.324
**Clinical outcome**				
Pneumatocele	3/140 (2.1)	31/96 (32.3)	34/236 (14.4)	<0.001
Bronchopleural fistula	1/140 (0.7)	13/96 (13.5)	14/236 (5.9)	<0.001
Tube insertion	29/140 (20.7)	18/96 (18.8)	47/236 (19.9)	0.743
Fibrolytics ^$^	12/140 (8.6)	14/96 (14.6)	26/236 (11)	0.203
VATS	1/140 (0.7)	42/96 (43.8)	43/236 (18.2)	< 0.001
Lung resection	1/140 (0.7)	15/96 (15.6)	16/236 (7.8)	< 0.001
Hospital day	8.47 ± 4.01	15.52 ± 6.91	11.32 ± 6.41	< 0.001

Data are presented as median ± SD, n/N (%), and No. (%), unless otherwise indicated.

CRP = C-reactive protein; VATS = video-assisted thoracoscopic surgery

^#^ Unknown/*Pneumococcus pneumoniae*/*Mycoplasma pneumoniae*/*Staphylococcus aureus*/others

* Comparing with (LUS) and (LUS + CT) groups

^$^ Treatment with intrapleural fibrolytics

### Correlation and degree of agreement between lung ultrasonography and computed tomography findings

**Fig 3:**
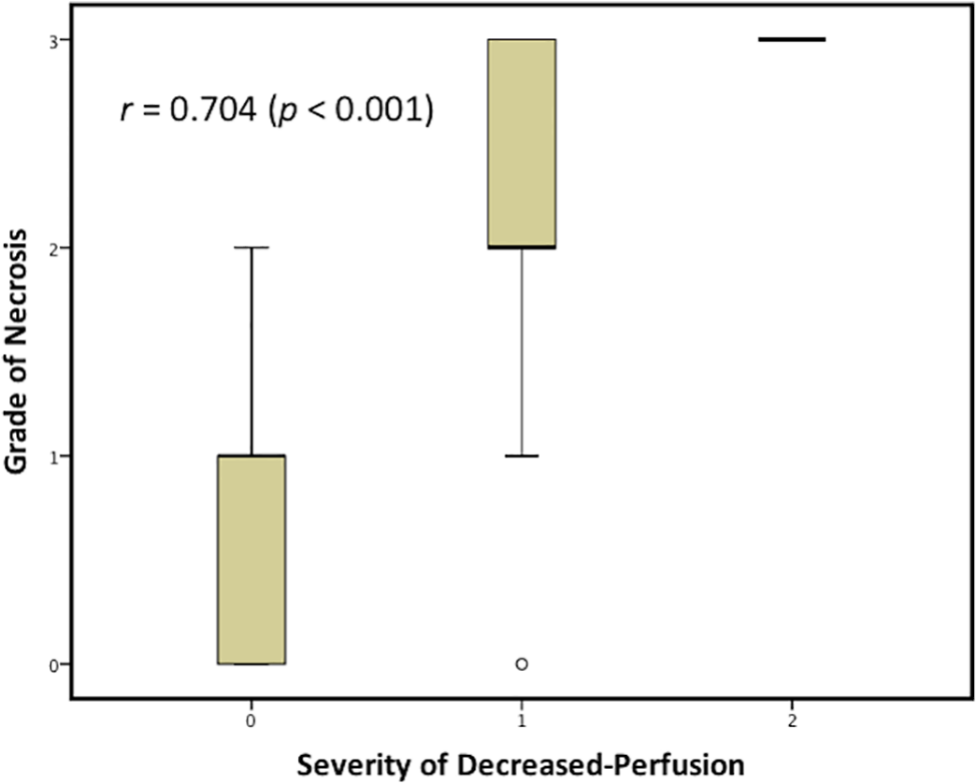
Spearman correlation between lung ultrasonography and computed tomography findings.

To evaluate the severity of lung necrosis, we assessed the CT findings on the LUS + CT group. In total, 24 cases (25%) had no necrotic lesions, 27 cases (28.1%) had minimal necrosis, 21 cases (21.9%) had moderate necrosis, and 24 cases (25%) had severe necrosis. LUS image results of the same patient population showed 54 patients (56.2%) having normal perfusion (P0), 31 patients (32.3%) having decreased perfusion (P1), and 11 patients (11.5%) having poor perfusion (P2). A significant dependence was observed after evaluating the degree of perfusion impairment and the severity of necrosis (Table 2). On further analyzing the correlation, a significantly positive correlation was estimated (*r* = 0.704, Fig 3).

**Table 2:** Contingency table of lung ultrasonography and computed tomography features.

	NP severity				
**Perfusion degree**		N0 (n = 24)	N1 (n = 27)	N2 (n = 21)	N3 (n = 24)
	P0 (n = 54)	23	22	9	0
	P1 (n = 31)	1	3	12	15
	P2 (n = 11)	0	2	0	9

*p* < 0.00001 analyzed using a chi-squared test.

P0 = normal perfusion; P1 = decreased perfusion; P2 = poor perfusion; HL0 = no hypoechoic lesion; N0 = no necrosis; N1 = minimal necrosis; N2 = moderate necrosis; N3 = massive necrosis

Ultrasonographic findings on HLs in the lung parenchyma have been reported to present in patients with NP [18]. Therefore, we also evaluated the incidence of HLs in our patient population. The results showed that 24.2% (17/70) of children among patients with NP in the LUS + CT group had HLs.

**Fig 4:**
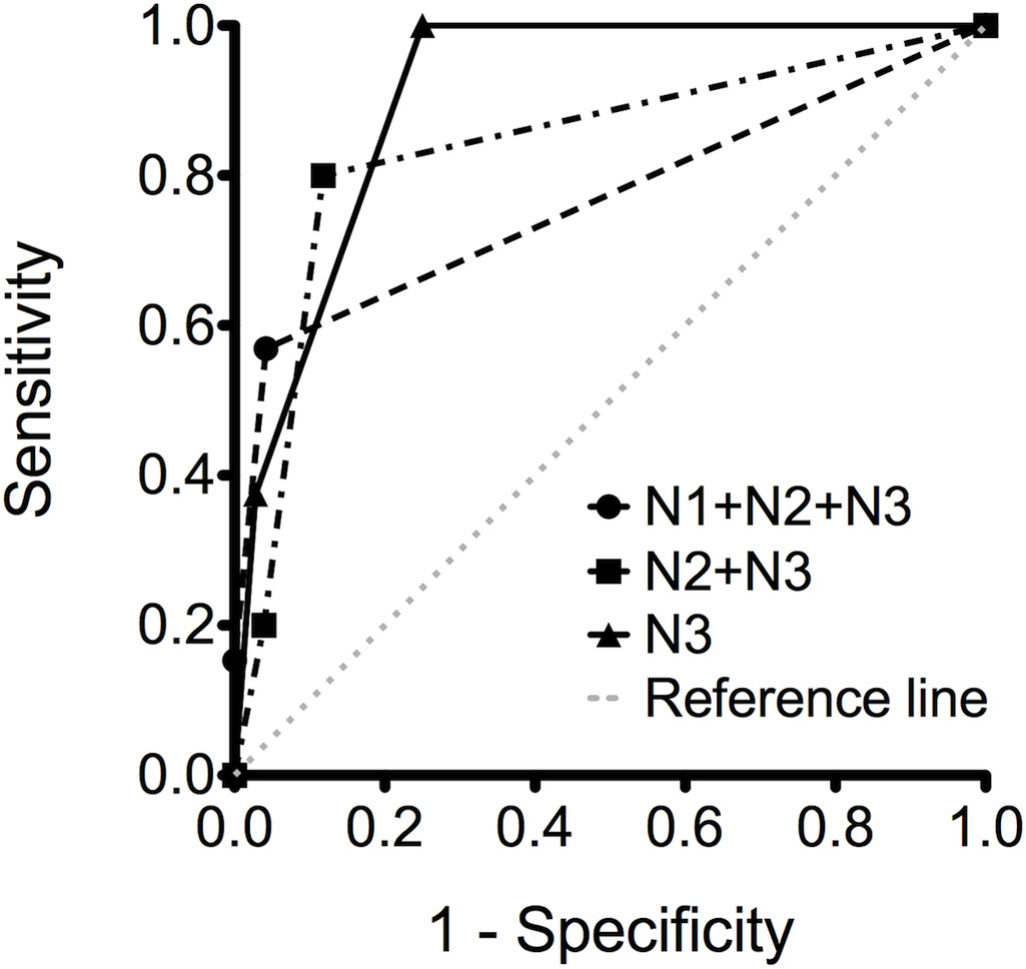
Receiver operating curves exhibiting impaired perfusion as a predictor of the varying severity of necrosis. N1 + N2 + N3 = minimal-to-massive necrosis; N2 + N3 = moderate-to-massive necrosis; N3 = massive necrosis.

Using the impairment of perfusion and HLs as the predictors of the severity of NP, we determined that both P2 and HLs had higher positive predictive values for the varying severity of necrosis (Table 3). Fig 4 shows the receiver operating characteristic curves describing the relationship between the sensitivity and specificity of changes from using the diverse degree of impaired perfusion for the diagnosis of the varying severity of NP. For predicting mild-to-massive (N1 + N2 + N3), moderate-to-massive (N2 + N3), and massive necrosis (N3), the areas under the curve were 0.767, 0.837, and 0.908, respectively.

**Table 3:** Ultrasonographic findings as predictors of the varying severity of necrotizing pneumonia.

NP Severity	Predictors	Sensitivity	Specificity	Positive Predictive Value	Negative Predictive Value
	*Percent*				
N1+N2+N3 (n = 72)	P1+P2	56.9	95.8	97.6	42.6
	P2	15.3	100	100	28.2
	HL	23.6	100	100	30.4
N2+N3(n = 47)	P1+P2	80.0	88.2	85.7	83.3
	P2	20.0	96.1	81.8	57.6
	HL	33.3	96.1	88.2	62.0
N3(n = 24)	P1+P2	100	75	57.1	100
	P2	37.5	97.2	81.8	82.4
	HL	50	93.1	70.6	84.8

P1 + P2 = decreased-to-poor perfusion; P2 = poor-perfusion; N1 + N2 + N3 = minimal-to-massive necrosis; N2 + N3 = moderate-to-massive necrosis; N3 = massive necrosis; HL = hypoechoic lesion

### Clinical outcomes of various lung ultrasonography findings

Children with CAP who exhibited impaired perfusion (P1or P2) in the LUS examination experienced longer periods of hospital stay, a higher incidence of pneumatocele formation, and an increased risk of requiring surgical lung resection (including wedge lung resection, segmentectomy, and lobectomy) (Table 4). Poor perfusion (P2) induced a higher occurrence of lung resection rescue therapy than did decreased perfusion (P1) (66.7% vs. 18.4%, *p* < 0.01). Patients with HLs in LUS also presented with poorer clinical outcomes, similar to those with CAP with impaired perfusion.

**Table 4:** Clinical outcomes related to various lung ultrasonographic findings.

LUS Findings (n = 236)	Hospital day	Pneumatocele	Lung resection
P0 (n = 186)	9.9 ± 5.0	11/186 (5.9)	1/186 (0.5)
P1 (n = 38)	16.1 ± 8.8*	17/38 (45)*	7/38 (18.4)*
P2 (n = 12)	18.1 ± 6.2*	6/12 (50)*	8/12 (66.7)* ^$^
HL0 (n = 216)	10.5 ± 5.3	21/216 (9.7)	8/216 (3.7)
HL (n = 20)	19.7 ± 10.6^#^	13/20 (65)^#^	8/20 (40)^#^

Data are presented as median ± SD and n/N (%).

P0 = normal perfusion; P1 = decreased perfusion; P2 = poor perfusion; HL0 = no hypoechoic lesion; HL = hypoechoic lesion

* Comparing with P0 (*p* < 0.001)

^#^ comparing with HL0 (*p* < 0.001)

^$^ comparing with P1 (*p* < 0.01)

### Prediction of clinical outcomes by using lung ultrasonography findings

Because more severe clinical manifestations and poorer outcomes were observed in the LUS + CT group (Table 1), analyzing only the LUS predictive values in the LUS + CT group would result in a significant bias. Therefore, we enrolled all cases, the LUS and LUS + CT group, for further investigation. To evaluate the accuracy of LUS in predicting the clinical outcomes of children with NP, the sensitivity, specificity, predictive values, and likelihood ratio of LUS findings in predicting the clinical consequence were analyzed (Table 5). After adjustment for the sex, age, leukocyte count, C-reactive protein level, pathogens, amount of effusion, and presence of coagulopathy or shock, both impaired perfusion and HLs could favorably predict subsequent pneumatocele formation and surgical lung resection (Table 5). The presence of impaired perfusion in LUS indicated a higher risk of requiring lung resection compared to the HL finding. Additionally, by combining both risk factors (impaired perfusion and hypoechoic lesion), far superior likelihood and odds ratios (ORs) were obtained for predicting pneumatocele formation. Moreover, the C-reactive protein level was an independent risk factor for pneumatocele formation (OR, 1.006; 95% CI, 1.001–1.012). By contrast, age is a protective factor against the development of pneumatocele formation and the requirement for surgical lung resection (OR, 0.715; 95% CI, 0.522–0.981; and OR, 0.446; 95% CI, 0.258–0.771, respectively).

**Table 5:** Ultrasonographic findings as predictors of clinical outcomes.

Clinical Outcome	Predictor	Sensitivity ^a^	Specificity ^a^	Positive Predictive Value ^a^	Negative Predictive Value ^a^	Positive Likelihood Ratio	Negative Likelihood Ratio	Odds ratio (95% CI)*
Pneumatocele	P1+P2	67.6	86.6	46	94.1	5.05	0.39	9.46 (3.49–25.61)
	HL	38.2	96.5	65	90.3	10.9	0.64	9.43 (2.81–31.62)
	P1+P2 and HL	38.2	97.3	68.4	90.3	14.2	0.64	10.11 (2.95–34.64)
Lung resection	P1+P2	93.8	84.0	30	99.5	5.86	0.07	40.86 (4.61–361.64)
	P2	98.2	50	66.7	96.4	1.96	0.04	85.64 (9.40–780.52)
	HL	50	94.5	40	96.3	9.09	0.53	8.19 (1.84–36.48)
	P1+P2 and HL	50	95	42.1	96.3	10	0.53	8.28 (1.86–36.93)
Hospital day >18 days	P1+P2 and moderate-to massive effusion	36.7	86.8	28.9	90.4	2.78	0.73	3.08 (1.15–8.29)

P1 + P2 = decreased-to-poor perfusion; P2 = poor-perfusion; HL = hypoechoic lesion

^a^ Presenting as percentage.

* For analyzing the odds ratio for pneumatocele and surgical resection, the sex, age, leukocyte count, C-reactive protein level, pathogens, amount of effusion, and presence of coagulopathy or shock in initial presentation were adjusted. For analyzing the odds ratio for the duration of hospitalization, the sex, age, leukocyte count, C-reactive protein level, pathogens, and presence of coagulopathy or shock in initial presentation were adjusted.

Regarding the duration of hospitalization, neither impaired perfusion nor HLs alone could predict longer hospitalization. However, the pattern obtained by combining the risk factors for impaired perfusion and effusion amount could predict a longer hospital stay (more than 18 days). In addition, the presence of shock during initial presentation was the most critical risk factor for the prediction of longer hospitalization (OR, 14.071; 95% CI, 2.611–75.843).

## Discussion

Based on our research, this is the first study to evaluate the correlation between LUS and the CT imaging of pediatric CAP with necrotizing changes. In addition, this is the first study to identify the role of LUS in predicting clinical outcomes. The results show a significant correlation between the degree of impaired perfusion in LUS and the severity of necrosis in CT. Furthermore, the degree of impaired perfusion can offer the highest sensitivity and specificity in recognizing massive necrotizing changes in the lung. By using LUS features as the predictors of clinical outcomes, massive lung necrosis can be easily predicted and the chance of pneumatocele formation and rescue lung-resection in children with CAP can be estimated.

The exact incidence of NP in pediatric CAP is unknown. In a large adult series of pneumococcal pneumonia, the incidence of necrotizing changes was estimated at approximately 6.6% [21]. However, NP has been increasingly recognized among the complications of pediatric CAP [1, 22]. The spectral shift of pneumococcal strains after the introduction of the pneumococcal vaccine [1, 4, 22, 23], the emergence of methicillin-resistance *S*. *aureus*, and the global usage of chest CT with early detection rates can all contribute to the rising incidence of NP [4, 24]. In this retrospective study, the occurrence of NP was as high as 33.9% when cases were diagnosed on the basis of necrosis recognized using chest CT. However, the incidence was as low as 14.4% when NP was identified by the later appearance of pneumatocele or bronchopleural fistula.

The necrotic foci of pneumonia without pleural involvement typically evolve into discrete pneumatocele formations [25]. If visceral pleura are involved in the necrotizing process, bronchopleural fistula occurs. The incidence of bronchopleural fistula in NP varied from 12% to 66% [1, 26].The low incidence of bronchopleural fistula (17.5%) observed in this study might be attributed to the early diagnosis of NP, fewer cases with staphylococcal infection, and a different spectrum of pneumococcal serotypes in Taiwan [22].

In complicated NP, massive necrosis and loss of blood supply result in persistent sepsis that is unresponsive to medical treatment. Massive necrosis can trigger intravascular coagulation and result in gangrenous changes to the involved area. Persistent bronchopleural fistula, a common complication of NP, often incurs obstinate air leakage and severe pneumothorax. In both situations, surgical intervention involving wedge resection/segmentectomy/lobectomy/pneumoectomy and the repair of bronchopleural fistula were advocated in some research [27–30]. Our previous study revealed that approximately 90% of children with NP exhibited the complication of bronchopleural fistula, and surgery revealed the pathological findings on pulmonary gangrene [22]. Similar to that study, among the patients who underwent lung resection in the current research, 68.8% (11/16) had persistent bronchopleural fistula and 37.5% (6/16) had pulmonary gangrenous changes. In the current study, the rate of surgical lung resection in patients with NP, 20%, was relatively low compared with that in previous studies on children and adults with NP [7, 31]. This was probably due to the concerns of respiratory function impairment following surgery, because the decision of lung resection still remains controversial in the pediatric population [32].

The early detection of NP most often depends on the results of a chest CT scan. In contrast-enhanced CT imaging, the presence of a poorly perfused area residing within the consolidated lung tissue remains the principal characteristic of necrotizing changes. In our unpublished data, the severity of necrosis in CT imaging correlated positively with rescue lung resection and longer hospitalization. Because a consolidated lung tissue is highly conspicuous in ultrasound, LUS can easily identify tissue liquefaction after necrosis (HL). By combining LUS with color Doppler, recognizing hypoperfused regions and detecting necrotizing changes early in patients with pneumonia is possible. Both HLs and the absence of vascularity in the consolidated lung have been described as major findings on NP [17, 18]. In the current study, the degree of impaired perfusion closely related with the severity of lung necrosis. Furthermore, by using the model of receiver operating curve, we revealed that the degree of impaired perfusion is a favorable predictor of massive necrosis in CT. However, our data disclosed that LUS offered low sensitivity for identifying mild-to-moderate necrosis. Because the LUS findings demonstrated a high likelihood ratio in predicting pneumatocele formation and poor clinical outcomes, the finding on specific characteristics of LUS (such as decreased-to-poor perfusion or HLs) indicates massive necrosis in the chest CT survey and portends poor outcomes. By contrast, the absence of these LUS features, especially that of decreased-to-poor perfusion, indicate the absence of NP in patients and a more favorable clinical outcome.

The present study has several limitations. First, this is a retrospective analysis of inpatient children who have been referred for the LUS analysis. Therefore, our study population might not have included a few children with CAP who did not have LUS performed. This selection bias might result in a mild overestimate of the prevalence of NP and overinflate the predictive value of LUS for lung necrosis. In addition, the decision of later intervention and management might be partly biased by the initial finding of the imaging study. However, the bias of clinical decision may be minimized since all examinations of LUS were performed early (within 2–3 days of admission). So LUS can still offer valuable information for predicting the clinical outcomes of CAP. Second, to analyze the correlation between LUS and CT findings, the standard approach is to perform both examinations simultaneously. Therefore, to minimize the predisposition, only cases who underwent CT within 3 days after LUS examination were enrolled for comparison. The CT and LUS images were assessed blindly. However, the result might still not offer real-time image concordance between CT and LUS. Third, because of ventilation–perfusion matching, a regional reduction of blood flow might often occur in a consolidated lung unit. This phenomenon occurs because of pulmonary vasoconstriction in response to hypoxia, which is regarded as a physiological process for preserving systemic oxygenation [33]. Thus, in our study, determining whether the reduced perfusion in LUS was due to lung necrosis or hypoxic vasoconstriction was not possible. Moreover, the development of collateral circulation within the necrotizing area can mislead the LUS interpretation of evolving necrosis, which mainly depends on the characteristic findings on hypoperfusion. Although both factors might reduce the specificity and sensitivity of LUS in the detection of necrotizing changes in CAP, repeated LUS evaluations might be able to reduce imprecisions and capture the emergence of NP early.

In conclusion, this study shows a high correlation between LUS and CT features in recognizing necrotizing changes in pediatric CAP, especially regarding massive necrosis. The LUS findings were also useful in predicting poor clinical outcomes. For the diagnosis of NP, LUS offers several benefits over chest CT, such as simple use and wide availability, no need for sedation in young patients, low cost, and no radiation exposure. On the basis of the findings of this study, we recommend the routine application of LUS in children with severe CAP. The appearance of certain LUS characteristics (impairment of perfusion or foci of HLs) is predictive of massive necrosis in the lung, and allows clinicians to make rapid therapeutic decisions and prompt early intervention therapy.
